# Feasibility of Using Daily Home Gait Testing to Assess Intervention Effectiveness in Older Adults

**DOI:** 10.1093/geroni/igaf122.116

**Published:** 2025-12-31

**Authors:** Pei-An Lee, Juhi Digvijay Salecha, Brad Manor, On-Yee Lo

**Affiliations:** Hebrew SeniorLife & Harvard Medical School, Boston, Massachusetts, United States; Hebrew SeniorLife & Harvard Medical School, Boston, Massachusetts, United States; Hebrew SeniorLife/Harvard Medical School, Boston, Massachusetts, United States; Harvard Medical School/ Marcus, Hebrew SeniorLife, Boston, Massachusetts, United States

## Abstract

Interventions focused on improving gait performance in older adults typically assess gait within single study visits at baseline and after intervention. This approach fails to account for non-trivial, within-subject daily fluctuations in gait performance. We previously validated a smartphone application (Gait App) enabling older adults to self-administer normal and ‘dual-task’ gait assessments in their own homes. This study examined the feasibility of deploying the Gait App to complete daily gait assessments both during and after a two-week intervention of noninvasive brain stimulation (10, once-daily stimulation sessions over two consecutive weeks) designed to improve dual-task gait in older adults. We enrolled 15 individuals at risk of falls (79±3yrs; 4 females; fear of falling via FES-I: 18-41; SPPB: 4-11). Participants were instructed to utilize the Gait App to complete a brief normal and dual-task gait assessments once a day, at least five days each week, throughout the intervention and over a subsequent 20-day follow-up. Participants successfully completed the assessment on 8.3±3.6 days (83% adherence) during the intervention phase and 11.3±13.7 days (57% adherence) throughout the follow-up period. Participants rated their experience with the Gait App on a scale from 0 (very poor) to 10 (very good), with an average score of 7.4. Six participants gave ratings of 9 or 10. These results demonstrate that the Gait App is feasible for monitoring daily gait performance under both normal and dual-task conditions in older adults at risk of falls, thereby enabling longitudinal assessment of how interventions impact mobility changes in older adults.

